# Subjective disgust and facial electromyography responses towards unedited and morphed overweight self‐pictures in women with varying levels of eating disorder symptomatology

**DOI:** 10.1002/erv.2940

**Published:** 2022-08-05

**Authors:** Irina Masselman, Peter J. de Jong, Klaske A. Glashouwer

**Affiliations:** ^1^ Department of Clinical Psychology and Experimental Psychopathology University of Groningen Groningen Netherlands; ^2^ Department of Eating Disorders Accare Child and Adolescent Psychiatry Groningen Netherlands

**Keywords:** anti‐fat bias, body dissatisfaction, eating disorder, facial EMG, pro‐thin bias, self‐disgust

## Abstract

Individuals with an eating disorder (ED) often report to be disgusted by their body. Body‐related self‐disgust could play an important role in the development and maintenance of EDs. We investigated if women with relatively high ED symptom scores indeed respond with disgust upon exposure to their body as indexed by facial electromyography (fEMG) of the m. levator labii superioris and self‐report. Given that one's self‐disgust may increase/decrease depending on the relative distance of the own body to the thin ideal, we also assessed women's disgust for overweight‐ and thin‐morphs of their body. Female undergraduate students (*N* = 104) were photographed and presented with their (morphed) body pictures, next to disgust‐relevant and overweight body control pictures. Higher levels of ED symptoms were associated with stronger self‐reported disgust to unedited body‐pictures and overweight‐morphs. Disgust to thin‐morphs was unrelated to ED symptoms. Participants generally showed heightened facial disgust towards overweight morphs, yet the strength of facial disgust was unrelated to ED symptoms. Thus, the findings provide evidence for the involvement of heightened body‐related self‐disgust in ED symptomatology, albeit only on the basis of self‐report.

## Key points

- Eating disorder symptoms were related to stronger subjective disgust to unedited and morphed overweight self‐pictures.
- This relationship was not evident for morphed underweight self‐pictures.
- Eating disorder symptoms were not associated with stronger fEMG activation to unedited and morphed self‐pictures.

## 1 INTRODUCTION

Eating disorders (EDs) are associated with a high mortality rate (Arcelus et al., 2011) and a poor quality of life (Jenkins et al., 2011). Despite this, individuals with an ED are often reluctant to seek professional help for their disordered eating (Eisenberg et al., 2011; Hudson et al., 2007). From the patients who do enter treatment, a considerable proportion does not recover or relapses after having successfully completed treatment (Berkman et al., 2007; Richards et al., 2000). In order to break the often chronic course of EDs and effectively address the invalidating consequences for both the patients and their family members, it is necessary to increase insight in the factors that promote the persistence of ED behaviour. Over recent years, theoretical models of EDs have increasingly recognized that disgust (e.g., towards food) and its underlying mechanisms (e.g., disgust conditioning) could be critically involved in the development and maintenance of eating pathology (Anderson et al., 2021; Hildebrandt et al., 2012; Troop & Baker, 2009).

Given that disgust is thought to have developed as a food rejection response (Rozin & Fallon, 1987), it is not surprizing that researchers have begun to study if the emotion of disgust underlies the persistent avoidance of food in EDs (Davey et al., 1998; Griffiths & Troop, 2006; Harvey et al., 2002; Troop et al., 2000, 2002). The disgust responses of individuals with an ED may, however, not be limited to food‐related cues but may also be elicited by the self (Espeset et al., 2012; Fox, 2009; Ille et al., 2014; Powell et al., 2015; Troop & Baker, 2009). It has been proposed that such self‐directed disgust could be an important factor in the development and persistence of EDs (Espeset et al., 2012; Fox & Power, 2009; Glashouwer & de Jong, 2021), although it is still up for debate how it exactly contributes to eating pathology. Given that EDs are associated with a tendency to judge self‐worth largely, sometimes even entirely, on the basis of the shape and weight of the own body (Fairburn et al., 2003), the physical self in particular (i.e., the own body) may be an important trigger of self‐disgust. For instance, disgust might be elicited by the experience of the own body as “too fat” according to societal appearance norms. Given that disgust sets off a strong, irrepressible urge to remove oneself from its elicitor (Rozin et al., 2000), individuals may try to escape the disgust towards their body by restricting their food intake, purging, exercising, or taking laxatives/diuretics. However, once acquired disgust associations have been found resistant to change (Bosman et al., 2016; Mason & Richardson, 2010; Olatunji et al., 2007). The self‐directed disgust reactions may thus persist after therapeutic interventions, which could explain why EDs are so persistent and why relapse is common among individuals in remission of an ED.

To date, there are only a handful of studies that examined the relationship between self‐disgust and eating pathology. Results from a qualitative study indicated that women with anorexia nervosa (*N* = 14) felt disgust when they were reminded of the appearance of their body and that these feelings of self‐disgust triggered body avoidance as well as a strong urge to lose weight and remove food from one's stomach (Espeset et al., 2012). Several quantitative studies further showed that women with high levels of eating pathology or a diagnosed ED scored relatively high on a self‐disgust questionnaire compared to women with low levels of ED symptoms (Chu et al., 2015; Olatunji et al., 2015) or without an ED (Ille et al., 2014). These elevated levels of self‐disgust have been found both for women with a (self‐identified) diagnosis of anorexia nervosa and bulimia nervosa (Bell et al., 2017). In addition, Bornholt et al. (2005) showed that adolescent girls with anorexia nervosa selected more disgust‐related words to describe their feelings when imagining a situation in which they had to focus on their body than underweight and overweight peers without an ED.

Previous research examining the role of self‐disgust in EDs has two limitations. First, so far no one has looked at whether the physical representation of oneself (i.e., the own body) evokes disgust, or whether the disgust of women with ED symptoms is in relation to the mental representation of the own body or the body‐concept. It is therefore unclear if women with ED symptoms experience disgust when being confronted with their physical self (e.g., when looking at a photo, mirror, or visible body‐parts). Second, except for the study of Bornholt et al. (2005), all previous studies relied on retrospective reports on emotional experiences. Since difficulties in monitoring emotions are common, especially among women with high levels of ED symptoms (Westwood et al., 2017), these retrospective reports may not provide an accurate picture of women's body‐related emotions. To adequately test whether exposure to the physical‐self elicits disgust, it would be important to assess women's reactions to their bodies in real‐time. This will also make it possible to assess if body‐related self‐disgust is expressed physiologically, for instance, by the typical facial expression of disgust. Undesirable bodies of others (i.e., emaciated and overweight) have been found to elicit a disgusted facial expression in women similar to that elicited by prototypical disgust stimuli (e.g., bodily products; de Jong et al., 2002; Dodd et al., 2017; Vartanian et al., 2018). An advantage of such an indirect/implicit measure of disgust is that it may be less sensitive to difficulties in assessing one's own emotions as well as socially desirable responding.

The present study was designed to overcome these limitations and more concretely test if body‐related self‐disgust is related to ED problems. More specifically, we tested if higher levels of ED symptoms were indeed associated with stronger disgust during exposure to pictures of the own body. In this study we not only relied on a self‐report measure of disgust but also included electromyography (EMG) activity of the levator labii superioris muscle as an index of facial disgust (Vrana, 1993). Given that the level of body‐related self‐disgust may increase/decrease depending on the relative distance of the own body to the sociocultural thin ideal (Fallon, 1990; Striegel‐Moore et al., 1986), we also examined the relationship between ED symptoms and women's disgust for overweight‐ and thin‐morphs of their body. Dodd et al. (2017) found that higher ED symptom levels were associated with more negative ratings of overweight female bodies. There was no relationship between ED symptom levels and the strength of facial disgust to the overweight bodies of others, however, this may be different when it comes to the own body. Women with an ED often have a very critical view of the own body (weight) but not of that of others (Voges et al., 2018), and previous research has indicated that for women with an ED there is a close link between ‘feeling fat’ and experiencing disgust (Espeset et al., 2012). The disgust in individuals with high levels of ED symptoms may thus be even more pronounced when confronted with overweight‐morphs of their body. Given the importance of thinness in the self‐evaluations of women with an ED (Fairburn et al., 2003), the disgust response may be attenuated or even absent when confronted to thin‐morphs of their body. Alternatively, it could be that individuals with high levels of ED symptoms show disgust upon confrontation with their thin‐morphs as well, considering that disgust‐associations have been found to be quite robust (Bosman et al., 2016; Mason & Richardson, 2010; Olatunji et al., 2007).

In short, we tested the following hypotheses: higher levels of ED symptoms are associated with (i) higher disgust ratings and (ii) stronger EMG activity in response to unedited pictures and overweight‐morphs of participants' bodies. In addition, we explored the relationship between ED symptoms and disgust for thin‐morphs of participants' bodies. To conceptually replicate the finding that, independent of ED symptom levels, women show facial disgust to overweight bodies of others (Dodd et al., 2017), we also explored women's affective responses to obese control bodies.

## 2 METHOD

### 2.1 Participants

A total of 112 female undergraduate students (*M age* = 19.5, *SD* = 1.49) were recruited via the University of Groningen and participated in the study for course credits. The only inclusion criterion apart from gender, was self‐indicated proficiency in the English language. The majority of participants had a European nationality (*n* = 106). Data of eight participants were excluded prior to the statistical analyses due to technical issues related to the EMG measurements (*n* = 3), software‐related errors (*n* = 2), refusal to wear the standardized clothing (*n* = 2), and reporting not looking at the body pictures during the task (*n* = 1). The remaining sample consisted of 104 participants. This study was approved by the Ethical Committee Psychology of the University of Groningen under the code PSY‐1819‐S‐0294.

### 2.2 Materials

#### 2.2.1 Stimuli

**FIGURE 1:**
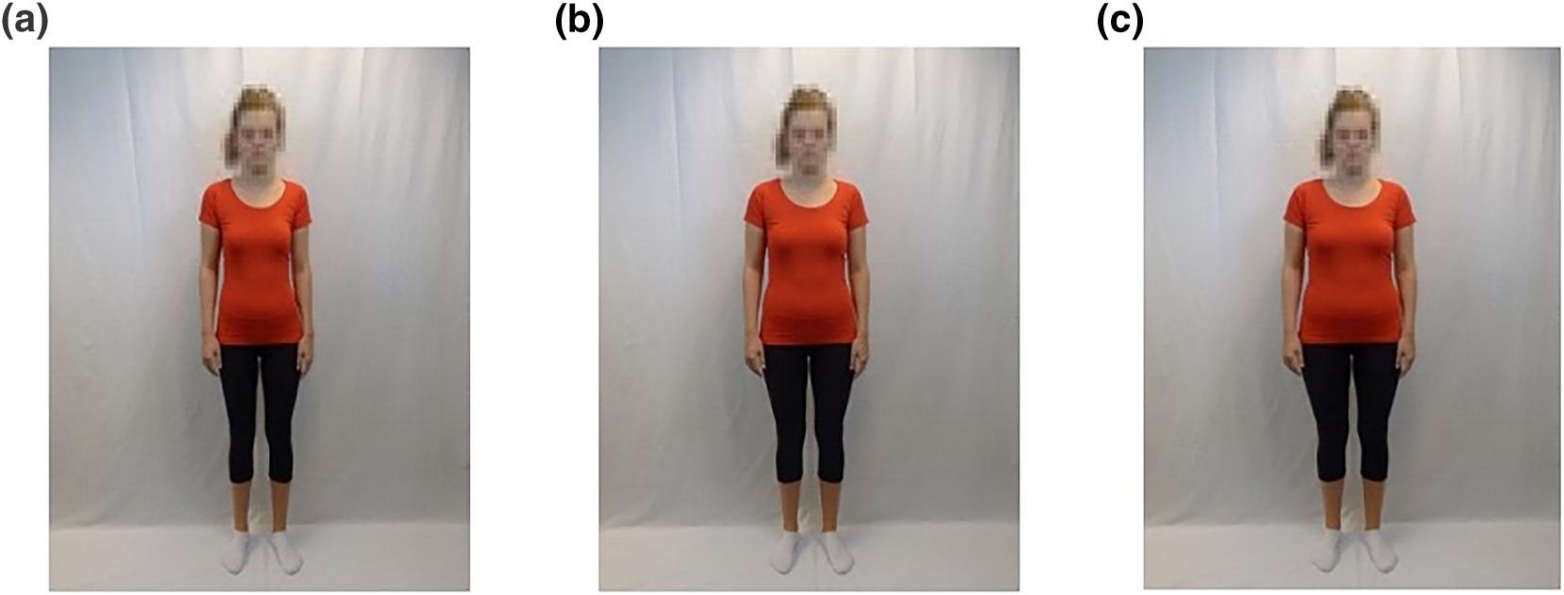
Example of a thin‐morph (a), unedited body‐picture (b), and overweight‐morph (the depicted person is not a participant but a volunteer model)

Nine pictures of the participants' body were used as stimuli (see Figure 1). Participants posed in standardized clothing (i.e., a black sports legging and a pink short‐sleeved T‐shirt) in front of a white background, while they held their arms next to their body and kept their facial expression neutral. A photograph was taken from the front, left, and right side of the body (with head), and the three unedited body‐pictures were cropped on the computer to 750 × 1000 pixels (i.e., width × length). To obtain three overweight‐morphs and three thin‐morphs, the unedited body‐pictures were edited using PortraitProBody3 software (Anthropics Technology, 2018). The pictures were adjusted one‐by‐one by selecting the maximum enlargement or reduction for the torso, arms, and legs.

To check the validity of the facial EMG (fEMG) recordings, we also included five pictures depicting pathogenic disgust elicitors (e.g., faeces and vomit) and five pictures that depicted threatening stimuli (e.g., a gun and fire) from the International Affective Picture System (IAPS; Lang et al., 1997). We also selected 25 neutral IAPS pictures (e.g., furniture and utensils). The majority of the neutral stimuli (*n* = 23) were validated in a previous study (*M* = 48.5, *SD* = 6.25, on a 100‐point valence scale; Masselman et al., 2022). In addition, we derived five pictures from the Internet of different morbidly obese female bodies (with clothes) with a nondescript or blurred background. The dimension of the affective and neutral pictures was 1024 × 768 pixels.

#### 2.2.2 Picture viewing task

The picture viewing task was programed using E‐prime 2.0 (Psychology Software Tools, 2002). We recorded fEMG during the picture viewing task to (implicitly) assess participants' spontaneous facial expressions in response to the stimuli. Before the task, participants were asked to relax for 2 minutes. After the 2 minutes, participants received written instructions that they had to focus on the centre of the screen and look at the picture that would sometimes appear. The stimuli were presented for 4 seconds against a light grey background. To oppose habituation, we used a variable inter‐stimulus interval (8, 10, or 12 s). During the inter‐stimulus interval a black fixation cross (59 × 60 pixels) was presented. Trials with a body picture or control stimulus (i.e., a disgust, threat, or overweight control body picture) were always preceded by a trial with a neutral stimulus. The nine (own) body pictures were presented first, and these pictures as well as the interposed neutral pictures were presented in the same order for all participants. After participants saw all body pictures of themselves, the affective stimuli were presented. The stimulus configuration for the affective stimuli (and interposed neutral stimuli) was completely random and the order of presentation thus differed across participants.

### 2.3 Measures

#### 2.3.1 ED symptoms

The severity of ED pathology was measured with the sixth version of the Eating Disorder Examination Questionnaire (EDE‐Q; Fairburn & Beglin, 2008). The EDE‐Q measures the presence of ED symptoms and body concerns in the last 4 weeks, and items are answered on a 7‐point Likert scale (0 = *not at all*/*no days* and 6 = *markedly*/*every day*). The total score of the EDE‐Q was used to assess participants' level of ED pathology (cf. recommendations of Aardoom et al., 2012). The total score can be calculated by averaging the scores of all items (except item 13 through 18), and a higher score indicates more severe eating pathology (*range* = 0–6). The EDE‐Q showed high internal reliability (Cronbach's *α* = 0.95).

#### 2.3.2 Affective ratings

All stimuli were first rated on valence (0 = *Very negative* and 100 = *Very positive*) and then on disgust (0 = *Not at all disgusting* and 100 = *Very disgusting*). The stimuli were presented one‐by‐one in the middle of a light grey computer screen with a Visual Analogue Scale (VAS) below. The stimuli were rated after (not during) the picture viewing task. Interim ratings might have led to awareness that the fEMG measured affective responses, and thereby might have reduced the genuineness of participants' facial expressions in response to the stimuli. The stimulus presentation schedule was consistent with the picture viewing task. The ratings were averaged per dimension for each stimulus type. Valence ratings were recoded (100—score) to facilitate interpretation.

### 2.4 Physiological responses

#### 2.4.1 fEMG recording

In accordance with recommendations (de Jong et al., 2002; Vrana, 1993; Wolf et al., 2005), fEMG activity was measured at the sites of the levator labii superioris and corrugator supercilii muscle. The latter is an index of emotional negativity and it was included to facilitate differentiation between disgust and global negative affective responding. We also measured fEMG activity at the site of the m. levator anguli oris and monitored participants' skin conductance response to the stimuli. Since these measures are not crucial for answering our hypotheses, we placed the analyses of these data in Appendix A (levator anguli oris) and B (skin conductance).

Pairs of Ag/AgCl electrodes (7 mm) were placed on the left side of the face (cf. the recommendations of Fridlund & Cacioppo, 1986). A single reference electrode was placed near participants' hairline above the nose. Before application, the electrodes were filled with conductance gel and participants' skin was cleaned with a cotton swab and Nuprep Skin Prep Gel (Weaver and company). Raw fEMG signals were sampled at a frequency of 2000 Hz, and Polybench software (hardware: TMSi Porti‐7, resolution = 0.0715 μV) was used to record the signal and synchronize it with the picture viewing task. The raw fEMG signals were processed offline with internally developed software called Aphys (version 5.1.0.0, Matlab version R2013a). Aphys processes the data in three steps (Step 1: bandpass filtering with finite impulse response (FIR) filter (28–950 Hz); Step 2: rectification; Step 3: smoothing with a lowpass FIR filter (40 Hz)) before giving the mean level of fEMG activity for a predefined epoch.

#### 2.4.2 fEMG data reduction

A stimulus‐dependent fEMG response was defined as the average change in activity in a muscle during the 4 seconds of stimulus presentation versus baseline (i.e., the average activity during the one second before stimulus onset). Thus, for each stimulus, we subtracted per muscle the average baseline activity from the average fEMG activity during stimulus presentation. fEMG responses were omitted if the mean activity during the baseline exceeded a threshold (*M*_*Baseline activity*_ + (3 × *SD*_*baseline activity*_)). The remaining fEMG responses were averaged per muscle and stimulus category. Higher m. corrugator supercilii activity was considered to capture a more global negative evaluation of the stimuli of that category, whereas higher m. levator labii superioris activity is taken to reflect stronger disgust (Vrana, 1993).

#### 2.4.3 Self‐disgust

For descriptive purposes, the Self‐disgust in Eating Disorders Scale (SDES; Moncrieff‐Boyd et al., 2014) was used as a trait measure of disgust directed towards the own body. It contains 16‐items that were answered on a 7‐point Likert scale (1 = *Strongly agree* and 7 = *Strongly disagree*). After recoding the reversed scored items and removing the six filler items, a sum score was calculated (*range* = 10–70). Higher scores indicate higher trait levels of subjective self‐disgust. The SDES showed a high internal reliability (Cronbach's *α* = 0.84).

#### 2.4.4 Disgust propensity and sensitivity

Propensity and sensitivity to feelings of disgust were measured for descriptive reasons with the revised Disgust propensity and sensitivity scale (DPSSR; van Overveld et al., 2006). Although the 16‐item version of the DPSSR was used, we omitted four items prior to our statistical analyses because a 12‐item version of the DPSSR has been shown to have superior psychometric properties (Fergus & Valentiner, 2009). This version of the DPSSR consists of six items that measure disgust propensity (i.e., how often an individual experiences feelings of disgust) and six items that measure disgust sensitivity (i.e., the extent to which these feelings are experienced as unpleasant). The items are scored on a 5‐point Likert scale (1 = *Never* and 5 = *Always*) and by summing the relevant items, a total score (*range* = 12–60) and subscale scores were calculated (*range* = 6–30). The DPSSR had an acceptable internal reliability (*α* = 0.75), and so did the propensity subscale (*α* = 0.73). The internal reliability of the sensitivity subscale (*α* = 0.65) was lower than that reported by previous studies (*range* = 0.77‐0.88; van Overveld et al., 2006, 2008; von Spreckelsen et al., 2018).

### 2.5 Procedure

All participants gave active informed consent. Participants were fully informed about the study procedure. However, we did not disclose that the electrodes measured their facial muscle activity as an index of their emotional reactivity. Participants were tested individually (duration = approximately 75 min). The experimenter estimated participants' clothing size. After posing for the pictures in the standardized clothing (size labels were covered), participants changed back into their own clothing and were seated behind a computer (1920 × 1080 IIYAMA, refresh rate = 60 Hz) to fill out the SDES, EDE‐Q, DPSSR, and some filler questionnaires on meaning in life (van Doornik et al., n.d) Next, the fEMG electrodes were attached to the participant. Participants then executed the picture viewing task and immediately afterwards rated the stimuli. After removing the electrodes, participants filled out several demographic questions and their height and weight (with clothes) were measured.

### 2.6 Statistical analyses

Six Pearson correlation coefficients were calculated between ED symptoms and self‐reported and physiological disgust for the three body stimuli types (i.e., unedited body‐pictures, overweight‐morphs, and thin‐morphs). In addition, similar correlational analyses were performed for the self‐report and fEMG measure of global negative affect. Alpha criterion was set to 0.008 (*α* = 0.05/6) for our primary analyses to correct for multiple testing.

## 3 RESULTS

### 3.1 Participants

For an overview of the means and standard deviations of the group characteristics, see Table 1. Height and weight measurements in the lab indicated that 17 participants had a BMI (kg/m^2^) that fell above what would be considered a healthy weight (i.e., BMI >25), whereas 4 participants were considered underweight (i.e., BMI < 18.5).^1^ BMI data of three participants were missing because of an error saving this information (*n* = 2) or because the participant refused to be weighed (*n* = 1). Inspection of the unedited body‐pictures of these participants did not indicate extreme BMIs, and the data of these participants were included in the (primary) analyses. Only one participant had a EDE‐Q total score above four, which is indicative for an ED. There was a moderate correlation between ED symptom severity and trait self‐disgust (as measured by the SDES; *r* = 0.40, *p* < 0.001). A total of 11 participants correctly indicated that the fEMG electrodes measured their facial expression in response to the stimuli.^2^

**TABLE 1:** Means and standard deviations for group characteristics

		*M (SD)*	*Range*
Descriptive variables	BMI	22.41 (2.69)	17.79–29.80
	EDE‐Q	1.69 (0.95)	0.15–5.09
	SDES	26.29 (8.66)	11.00–52.00
	DPSSR	30.21 (5.99)	15.00–46.00
	Propensity	17.25 (3.59)	9.00–25.00
	Sensitivity	12.96 (3.74)	6.00–24.00

Abbreviations: BMI, Body Mass Index (BMI = kg/m^2^); DPSSR, Total score on Disgust Propensity and Sensitivity Scale ‐ revised (range 12–60; higher scores indicate higher trait disgust); EDE‐Q, Eating Disorder Examination Questionnaire (range 0–6; higher scores indicate more severe body concern and eating pathology); Propensity, Propensity subscale of the DPSSR (range 6–30; higher scores indicate higher trait disgust propensity); SDES, Self‐disgust in Eating Disorders Scale (range 10–70; higher scores indicate more self‐disgust); Sensitivity, Sensitivity subscale of the DPSSR (range 6–30; higher scores indicate higher trait disgust sensitivity).

### 3.2 Validity fEMG recordings

Due to a right skew and many outlying datapoints (i.e., data points that extended 1.5 times the interquartile range), the validity of the fEMG measurements was tested non‐parametrically with four Wilcoxon Signed Ranks Tests. Compared to the neutral stimuli, participants responded to the pathogenic disgust stimuli with stronger m. levator labii superioris activity (*M*_*difference*_ = 1.28, *SD* = 2.99, *Z* (103) = −5.76, *p* < 0.001, *r* = 0.56) and m. corrugator supercilii activity (*M*_*difference*_ = 1.15, *SD* = 3.08, *Z* (103) = −6.22, *p* < 0.001, *r* = 0.61). Participants responded to the threat stimuli with stronger m. corrugator supercilii activity (*M*_*difference*_ = 0.34, *SD* = 0.88, *Z* (103) = −3.03, *p* = 0.002, *r* = 0.30), but the fEMG activity on the site of the m. levator labii superioris did not differ for the threat stimuli and neutral stimuli (*M*_*difference*_ = 0.09, *SD* = 0.79, *Z* (103) = −1.33, *p* = 0.18, *r* = 0.13). These results indicate that, compared to the activity elicited by the neutral stimuli, participants responded with stronger facial disgust to the pathogenic disgust stimuli and with stronger facial negative affect but not disgust to the threat stimuli. Thus, the fEMG measurements were sufficiently sensitive to measure and differentiate between global negative affect and disgust.

### 3.3 Hypotheses testing

Table 2 provides an overview of the means and standard deviations of participants' disgust responses to their body stimuli as well as the correlations between the indices of disgust and ED symptoms. Preliminary analyses indicated that the disgust data (self‐report and fEMG) were strongly right skewed and contained many outlying datapoints. Since visual inspection gave us no reason to assume that the outlying data were invalid measurements, the hypotheses were tested non‐parametrically using Spearman's rho correlation coefficients. Results indicated moderate positive relationships between ED symptoms and the self‐reported disgust for the unedited body‐pictures as well as the overweight‐morphs. The relationship between the level of ED symptoms and the strength of the self‐reported disgust for the thin‐morphs was not significant. In addition, there was no significant relationship between the level of ED symptoms and the magnitude of facial disgust as elicited by the unedited body‐pictures, overweight‐morphs, and thin‐morphs. Power analyses using G*Power 3.1 (Faul et al., 2009) indicated that our primary analyses had a power of 0.05, 0.68, and >0.99 to detect a small, medium, and large‐sized effect, respectively (*ρ* = 0.3, *α* = 0.008, two‐tailed).

**TABLE 2:** Summary of means, standard deviations, and correlation coefficients for eating disorder symptoms and disgust responses

	*M (SD)*	*Range*	*R*	*p*
Disgust rating				
Unedited body	26.53 (24.30)	0–89.00	0.48	<0.001^a^
Overweight‐morphs	47.43 (30.56)	0–99.67	0.45	<0.001^a^
Thin‐morphs	28.06 (23.32)	0–90.67	0.11	0.28
M. levator labii superioris				
Unedited body	0.38 (0.77)	0–5.78	0.04	0.70
Overweight‐morphs	0.64 (1.31)	0–8.77	0.17	0.08
Thin‐morphs	0.37 (0.67)	0–3.77	0.19	0.05

*Note*: Disgust rating (range 0–100, higher scores indicate more self‐reported disgust for the body pictures); m. levator labii superioris (in microvolts, higher scores indicate a stronger facial disgust response to the body pictures).

^a^ Significant at an alpha level of 008.

Additional analyses (Pearson correlations) indicated that ED symptoms were moderately positively associated with self‐reported negative affect for the unedited body‐pictures and overweight‐morphs (see Table 3). We also found a weak yet significant negative relationship between ED symptoms and self‐reported negative affect for the thin‐morphs. Due to a strong right skew and outliers, non‐parametric Spearman's rho correlation coefficients were calculated to assess the relationship between ED symptoms and negative affect to the (morphed) body pictures as indexed by fEMG activity of the m. corrugator supercilii. There was no significant relationship between the level of ED symptoms and the magnitude of facial negative affect to the unedited body‐pictures, overweight‐morphs, and thin‐morphs.

**TABLE 3:** Summary of means, standard deviations, and correlation coefficients for eating disorder symptoms and global negative affective responses

	*M* *(SD)*	*Range*	*r*	*p*
Negative affect rating				
Unedited body	50.89 (17.46)	0.33–91.00	0.43	<0.001^a^
Overweight‐morphs	71.26 (17.21)	3.00–99.67	0.43	<0.001^a^
Thin‐morphs	51.02 (20.42)	3.33–93.33	−0.29	0.002^a^
M. corrugator supercilli				
Unedited body	0.39 (0.66)	0–4.54	0.19	0.05
Overweight‐morphs	0.41 (1.00)	0–8.85	0.13	0.20
Thin‐morphs	0.42 (0.77)	0–3.79	−0.12	0.24

*Note*: Negative affect rating (range 0–100, higher scores indicate a more negative evaluation of the body pictures); m. corrugator supercilii (in microvolts, higher scores indicate a stronger facial negative affective response to the body pictures).

^a^ Significant at an alpha level of .008.

### 3.4 Overweight control bodies

To test whether women exhibit facial disgust when looking at pictures of overweight women (cf. Dodd et al., 2017; Vartanian et al., 2018), we planned to perform two paired‐sample *t*‐tests to compare fEMG in response to overweight control bodies versus neutral stimuli. However, due to outliers and a right skew of the data, two Wilcoxon Signed Ranks Tests were performed instead. Compared to neutral stimuli, the overweight control bodies elicited stronger m. corrugator supercilii activity (*M*_*difference*_ = 0.20, *SD* = 0.61, *Z* (103) = −2.48, *p* = 0.013, *r* = 0.24). The difference in m. levator labii superioris activity showed a non‐significant trend in the same direction (*M*_*difference*_ = 0.07, *SD* = 0.66, *Z* (103) = 1.76, *p* = 0.078, *r* = 0.17). Thus, on average, participants responded with facial global negative affect, but not disgust to pictures of overweight female bodies. The subjective ratings showed a similar pattern: Participants rated the overweight control bodies on average as slightly negative (*M* = 64.2, *SD* = 16.7), but not as very disgusting (*M* = 40.4, *SD* = 26.2). Consistent with the findings of Dodd et al. (2017), eating pathology did not significantly correlate (Spearman's rho) to activity in the m. levator labii superioris (*r* = 0.09, *p* = 0.36) or m. corrugator supercilii (*r* = 0.11, *p* = 0.28). Eating disorder symptoms did, however, show a (weak) significant correlation with self‐reported disgust (Spearman's rho: *r* = 0.21, *p* = 0.032), but not with self‐reported negative affect elicited by the overweight control stimuli (Pearson: *r* = 0.13, *p* = 0.20; note that these self‐report measures were not included in the study of Dodd et al., 2017).

## 4 DISCUSSION

We examined the relationship between self‐reported ED symptoms and women's subjective and physiological disgust for pictures of their body as well as for overweight‐ and thin‐morphed versions of these pictures. The major findings can be summarised as follows: (i) the level of ED symptoms was moderately positively correlated with the strength of self‐reported disgust for the unedited body‐pictures and overweight‐morphs; (ii) ED symptom severity was not associated with strength of facial EMG responses to the unedited body‐pictures and overweight‐morphs; (iii) ED symptom severity was not associated with the level of self‐reported disgust and facial EMG responses to the thin‐morphs.

### 4.1 Current body

Our findings showed that ED symptom levels were indeed positively related to disgust upon confrontation with the own (unedited) body. Yet, this relationship was restricted to the self‐report measure of disgust and not similarly evident for the fEMG activation of the m. levator labii superioris. ED symptom severity was also not associated with stronger activation of the m. corrugator supercilii. Thus, women with relatively high levels of ED symptoms did not respond to their own body with stronger facial expressions of disgust nor of global negative affect compared to women with relatively low levels of ED symptoms.

These findings indicate that disgust elicited by confrontation with the own body is experienced on a subjective level without giving concurrently rise to muscular activity that can result in the typical facial expression of disgust. This might indicate that self‐disgust is markedly different from other forms of disgust for which activation of the levator labii muscle is consistently found (e.g., Chapman et al., 2009). Perhaps, self‐disgust is not accompanied by a facial expression because it is disadvantageous to signal such feelings to others (e.g., because it increases the likelihood of social rejection). If so, self‐disgusted women may be highly motivated to suppress their facial reaction when seeing themselves. An alternative explanation for our findings is that other facial muscles are more important than the levator labii within the context of self‐disgust. For instance, disgust elicited by social and moral transgressions is associated with activation of the m. levator anguli oris (Rozin et al., 1994). Although we found no evidence that ED symptom levels were associated with levator anguli oris activation to the body stimuli (see Appendix A), it is still possible that we missed stimulus‐dependent fEMG activity because we restricted our measurements to only three facial muscles.

It could also be that our results underestimate the involvement of facial expressions in self‐disgust. First, the body stimuli may not have been disgusting enough to result in a physiological response. After all, the body stimuli were overall rated far less disgusting (unedited body‐pictures: *M* = 26.5, *SD* = 24.3; Overweight‐morphs: *M* = 47.4, *SD* = 30.6) than the pathogenic stimuli (*M* = 87.7, *SD* = 12.6) for which we did find fEMG responses. Second, the difference in findings between the self‐report and physiological measures may have been due to the latency of women's self‐disgust response. Individuals with high and low levels of ED symptoms may not differ in how they initially respond to their body (i.e., in the first 4 s), but for those with high levels of ED symptoms the own body pictures may have triggered additional evaluative processes that led to self‐disgust (disgust rating of body‐pictures on average took 6 s).

### 4.2 Overweight body

We found a positive relationship between ED symptom levels and subjective disgust to the overweight‐morphs, but no relationship between ED symptom levels and levator labii activation. The correlation between ED symptoms and subjective disgust was not stronger for the overweight‐morphs than for the unedited body‐pictures. Yet, it is important to note that the results from our exploratory analyses (Appendix C) indicated that all participants on average responded with stronger subjective and facial disgust to the overweight‐morphs than to the unedited body‐pictures. Thus, participants with high and low levels of ED symptoms showed a relatively similar increase in subjective disgust when an overweight version of their body was shown instead of their actual body. This is in support of the idea that body‐related self‐disgust increases when the body deviates more from the sociocultural thin ideal. However, contrary to expectations, this was a general phenomenon and not more pronounced in women with relatively high levels of ED symptoms. This is in line with previous research which found comparable levels of (implicit) anti‐fat bias in women with and without an ED (Behrens et al., 2021), and women with and without a restrictive diet (Vartanian et al., 2005). The finding that participants on average responded with stronger facial disgust to the overweight‐morphs than unedited body‐pictures seems also at odds with the idea that the overweight‐morphs were simply not disgusting enough to elicit a physiological response.

The levator labii activation to overweight versions of the own body does not seem to reflect a general response to overweight body shapes, considering that participants responded with self‐reported and facial negative affect but not disgust to the overweight bodies of control women. The latter also means that we were not able to conceptually replicate the finding of Dodd et al. (2017) that women show activation of the m. levator labii superioris when viewing overweight female bodies (although there was a non‐significant trend). Consistent with the findings of Dodd et al. (2017), ED symptom levels were not associated with the strength of levator labii (and corrugator) activation to the overweight control women. ED symptom levels were associated with self‐reported disgust to the overweight control women. A self‐report measure of disgust was not included in the study of Dodd et al. (2017), and more research is necessary to examine the robustness of this finding, particularly because the association between ED symptoms and disgust ratings was weak, and we failed to find a similar association between ED symptom levels and negative affect ratings. By and large, our findings suggest that negative attitudes towards fat bodies, both when it pertains to the self and others, are common in the general population, and are not an unique characteristic of individuals with ED symptomatology.

### 4.3 Thin body

Results from our exploratory analyses indicated that ED symptom severity was not associated with the level of subjective or facial disgust to the thin‐morphs. It could be that the stronger subjective disgust that women with higher levels of ED symptoms showed for their unedited body‐pictures and overweight‐morphs reduced (normalised) when thinner versions of the own body were presented. However, since we did not find any overall differences in how participants responded to the thin‐morphs and the unedited body‐pictures (Appendix C), this pattern of results seems to be at least partly driven by women with low levels of ED symptoms who responded negatively to the thin‐morphs. Higher levels of ED symptoms were associated with less self‐reported negative affect to thinner versions of the own body.

Together, our findings suggest that ED symptomatology is associated with a relatively positive evaluation of thinness. The present results are consistent with the view that self‐directed disgust is influenced by the level of deviation of the body from sociocultural weight ideals. Nevertheless, it seems relevant to consider that the contrast with the unedited body‐pictures and overweight‐morphs may have influenced how the thin‐morphs were evaluated. It is therefore unclear how these findings translate to a natural development where dieting might gradually result in a thinner body.

### 4.4 Strengths and limitations

The present study is the first to assess body‐related self‐disgust in real‐time using pictures of the own (morphed) body, and the first to incorporate fEMG measurements to index body‐related self‐disgust. There were, however, several limitations. First, the mean level of ED symptoms was relatively low in our sample. Certain procedural elements (e.g., being photographed) could have prevented women with severe ED symptoms from participating in this study. Consequently, our results may underestimate the strength of the association between eating pathology and facial disgust to the own (morphed) body. Second, we did not check if women were able to correctly identify their unedited body‐pictures. Body size overestimations are quite common among women (e.g., McCabe et al., 2006), and especially among women with an ED (Farrell et al., 2005). Third, with the available software it was not feasible to standardise the BMI of the morphs. Women with an unhealthy BMI could have inflated the results (because their morphs were more extreme) or, alternatively, could have reduced the effect sizes (because all body stimuli fell in the same BMI category). A next step in future work could be to determine if in individuals with more extreme levels of ED symptomatology or a clinically diagnosed ED elevated body‐related self‐disgust is also evident at the physiological level. Inclusion of a wider‐range of standardized morphs, and a check of whether women were able to recognise their actual body, could help to elucidate how changes in BMI affect body‐related self‐disgust. More research is also necessary to assess if our findings can be replicated in different populations (e.g., male, obese, or depressed), and to examine variables that may moderate/mediate the relationship between ED symptoms and body‐related self‐disgust (e.g., internalising symptoms, current BMI, or sexual orientation; Meneguzzo et al., 2022).

## 5 CONCLUSIONS

This study examined the relevance of body‐related self‐disgust in ED symptomatology and tested whether especially for those with high levels of ED symptoms exposure to pictures of the own body would elicit disgust, and whether this relationship would be especially pronounced when faced with overweight‐morphed self‐pictures. Although findings indicated that subjective and facial disgust responses to overweight versions of the own body represent a common phenomenon, the strength of the subjective disgust response to both overweight and unedited body‐pictures was stronger in those with relatively high levels of ED symptoms. By and large, the findings are consistent with the view that body‐related self‐disgust is involved in ED symptomatology, albeit that heightened self‐disgust was only evident at the subjective level.

## AUTHOR CONTRIBUTIONS

Irina Masselman: Conceptualization, Methodology, Software, Formal analysis, Investigation, Writing ‐ original draft. Klaske Glashouwer: Conceptualization, Methodology, Resources, Writing ‐ review & editing, Supervision, Project administration, Funding acquisition. Peter de Jong: Conceptualization, Methodology, Writing ‐ review & editing, Supervision.

## CONFLICT OF INTEREST

The authors declare that they have no conflicts of interest.

## PRE‐REGISTRATION

The present study is preregistered at https://aspredicted.org/ge2km.pdf. We deviated from the pre‐registered statistical plan because the planned analyses (multiple regressions) were inappropriate to test the pre‐registered hypotheses.

## Supporting information

Supporting Information S1

## Data Availability

The data that support the findings of this study are openly available in Dataverse NL at https://doi.org/10.34894/FCXDRF.

## ENDNOTE

^1^
When the primary analyses were repeated without the data of the overweight and underweight participants, there were no major changes in the results other than that the relationship between ED symptoms and self‐reported disgust for the thin‐morphs became a bit stronger (*r* = 0.17, *p* = 0.098).

^2^
Repeating the primary analyses without the fEMG data of these participants did not change the pattern of the results.
