# Supplementary material for: Subjective disgust and facial electromyography responses towards unedited and morphed overweight self‐pictures in women with varying levels of eating disorder symptomatology
Source: Eur Eat Disord Rev. 2022 Aug 5;31(1):98–109. doi: 10.1002/erv.2940 (PMC10087951; doi:10.1002/erv.2940)
Supplement: Supplementary file 1 — Supporting Information S1 [file ERV-31-98-s001.docx]

**Appendix A**

For exploratory purposes, we examined if high levels of ED symptoms were associated with stronger activation of the m. levator anguli oris in response to the own body-pictures and morphs. The m. levator anguli oris appears to be important in responding to social and moral transgressions (Rozin et al., 1994), and therefore may be particularly responsive to viewing the own body when this body is deemed in violation of societal appearance norms. The procedure of data collection and reduction was kept consistent with the other fEMG measures. Since the data contained many outliers, Spearman’s rho correlation coefficients were calculated instead of Pearson correlations. Results indicated that the level of ED symptoms was not associated with strength of the activity in the m. levator anguli oris upon confrontation with the unedited body-pictures (*M* = 0.55, *SD* = 0.86, *r* = -.05, *p* = .65), overweight-morphs (*M* = 0.82, *SD* = 1.61, *r* = .08, *p* = .40), or thin-morphs (*M* = 0.55, *SD* = 0.96, *r* = .10, *p* = .32). This is in line with the results of our primary analyses which similarly showed that ED symptom severity was not associated with the magnitude of facial disgust to the (morphed) body stimuli.

**Appendix B**

The skin conductance response (SCR) was included as an exploratory measure to assess the relationship between ED symptoms and participants’ sympathetic arousal in response to the own (morphed) body. An electrode (identical to those used to measure fEMG) was placed at the tip of the middle finger and ring finger of the participant’s non-dominant hand. The electrodes were filled with a TD-246-4 Skin Conductance Electrode Paste (Discount Disposables). Skin conductance was measured with a sampling frequency of 2000 Hz by an internally developed device, which uses a constant sinusoidal measuring voltage of 0.5 Hz. The signal was recorded by Polybench software (hardware: TMSi Porti-7, resolution = 1.4305 µV) and filtered with a lowpass FIR filter (10 Hz) to remove noise from the signal. A stimulus-dependent SCR was defined as the maximum increase in skin conductance (i.e., the amplitude of the SCR signal) during the one to four seconds after stimulus onset. Unlike the procedure for the fEMG data, we visually inspected the SCR data in Aphys and decided on a trial-by-trial basis if the data had to be omitted using the following criteria: i) Trials for which there was a negative SCR during the four-second post-stimulus epoch were labeled as non-response (i.e., the SCR was set to zero); ii) When the SCR started before or during the baseline, the trial was omitted. The SCRs were averaged per stimulus category, and these sampled values were divided by 10,000 to get the mean SCR in microsiemens. Higher SCRs indicate stronger physiological arousal in response to a stimulus.

*Validity SCR recordings*

Preliminary analyses indicated that the SCR data contained many outlying datapoints. Based on visual inspections, we concluded that the datapoints appeared to be valid measurements (i.e., the SCR signal was within the normal range and fluctuated per trial). Therefore, we tested the validity of the SCR measurements non-parametrically with two Wilcoxon Signed Ranks Tests. Results indicated that, compared to the neutral stimuli, participants did not respond with a stronger SCR to the pathogenic disgust stimuli (*M _difference_* = 0.01, *SD* = 0.15, *Z*(103) = 0.83, *p* = .41, *r* = .08), but they did respond with a stronger SCR to the threat stimuli (*M _difference_* = 0.08, *SD* = 0.24, *Z*(102) = -3.78, *p* < .001, *r* = .37). Thus, the SCR recordings were only sufficiently sensitive to pick up changes in sympathetic arousal in response to threat stimuli and not disgust stimuli.

*Results*

Given the assumption violations, Spearman’s rho correlation coefficients were calculated. The results indicated that ED symptoms were not associated with participants’ skin conductance in response to the unedited body-pictures (*M* = 0.18, *SD* = 0.25, *r* = .033, *p* = .74), overweight-morphs (*M* = 0.14, *SD* = 0.21, *r* = .05, *p* = .59), or thin-morphs (*M* = 0.16, *SD* = 0.23, *r* = .11, *p* = .27). Thus, higher levels of ED symptoms were not associated with higher levels of sympathetic arousal in response to the (morphed) body stimuli.

**Appendix C**

To check whether participants overall responded differently to the unedited body-pictures and morphs, 6 Wilcoxon Signed Ranks Tests were performed (because of the skewness of the data). The results indicated that there was no overall difference in how participants responded to the unedited body-pictures and the thin-morphs (Disgust rating: *M _difference_* = 1.54, *t*(103) = -0.67, *p* = .50; M. levator labii superioris: *M _difference_* = 0.01, Z(103) = -0.06, *p* = .95). However, participants did respond with more self-reported disgust and m. levator labii superioris activity to the overweight-morphs of their body compared to the unedited body-pictures (Disgust rating: *M _difference_* = 20.9, *t*(103) = -12.0, *p* < .001; M. levator labii superioris: *M _difference_* = 0.26, Z(103) = 2.18, *p* = .030) and compared to thin-morphs (Disgust rating: *M _difference_* = 19.4, *t*(103) = -7.22, *p* < .001; M. levator labii superioris: *M _difference_* = 0.26, Z(103) = -2.60, *p* = .009). Thus, participants responded with stronger facial disgust to the overweight-morphs, and rated them as more disgusting than the unedited body-pictures and thin-morphs. The self-reported and facial disgust for the thin-morphs was comparable to that for the unedited body-pictures.
